# Evaluating and Comparing Flexure Strength of Dental Models Printed Using Fused Deposition Modelling, Digital Light Processing, and Stereolithography Apparatus Printers

**DOI:** 10.7759/cureus.54312

**Published:** 2024-02-16

**Authors:** Noina Atwal, Deepankar Bhatnagar

**Affiliations:** 1 Orthodontics and Dentofacial Orthopaedics, Maharishi Markandeshwar College of Dental Sciences and Research, Maharishi Markandeshwar University, Ambala, IND

**Keywords:** 3d printing, dentistry, digital dentistry, sla, stereolithography apparatus, orthodontics, dlp, fdm, dental models, flexure strength

## Abstract

Introduction: The introduction of three-dimensional (3D) printing in dentistry has mainly focused on applications such as surgical planning, computer-guided templates, and digital impression conversions. Additive manufacturing (AM), also known as 3D printing, involves layering resin material sequentially to construct objects and is gaining recognition for its role in creating custom-made medical appliances. The field of orthodontics has also embraced this technological wave and with the advent of cost-effective printers and biocompatible resins, 3D printing has become increasingly feasible and popular in orthodontic clinics. The limitations of traditional plaster models may have prompted the emergence of 3D-printed models, but it led to enhancing treatment planning and device fabrication, particularly in orthodontics. Notable desktop printing technologies include fused deposition modelling (FDM), digital light processing (DLP), and stereolithography (SLA), each employing distinct methods and materials for fabricating appliances. Evaluating mechanical properties, like flexure strength, is crucial to determine the material's ability to withstand bending forces and thus prove useful in fabricating thermoformable appliances, surgical templates, etc. This study aims to assess the flexure strength of 3D-printed models using FDM, DLP, and SLA technology, providing insights into their suitability as replacements for conventional models and shedding some light on the durability and sustainability of 3D-printed models.

Materials and methodology: Cuboids measuring 20 x 5 x 2 mm were cut from models, creating 10 samples per printer group. These samples underwent flexure strength testing using a three-point bending system in a universal testing machine.

Results: The FDM group exhibited the highest flexure strength at 69.36 ± 6.03 MPa, while the DLP group showed the lowest flexure strength at 67.47 ± 20.58 MPa. The results can be attributed to the differences in resin materials used for fabrication, with FDM using acrylonitrile butadiene styrene (ABS) polymer and SLA/DLP using polymethyl methacrylate (PMMA), and also to the variation in their printing mechanism.

Conclusion: The findings affirm the suitability of FDM models for orthodontic applications, suggesting enhanced efficiency and reliability in clinical practices.

## Introduction

The adoption of digital technology witnessed a significant rise in the 1980s in the field of dentistry, particularly due to the amalgamation of computer-aided design and manufacturing (CAD/CAM). Initially, this technology transformed major industries like aviation and automobiles and later carved its place in medicine, enhancing the precision and safety of surgical procedures. Dentistry also became a part of this evolution, where it utilized three-dimensional (3D) printing for surgical planning, digital impression conversions, and computer-guided templates[1]. 3D printing, also known as additive manufacturing (AM), involves layering of the resin material sequentially to form objects, gaining distinction for its role in custom-made medical appliances. Chuck Hall in 1984 introduced stereolithography (SLA) technology making him the pioneer of this pivotal moment in the history of 3D printing [2].

While the "gold standard" of dental models has always been plaster, its limitations led to the disclosure of digital models produced through oral scanners. This technological wave was then embraced by orthodontics, taking a revolutionary leap from traditional methods. The introduction of cost-effective printers along with biocompatible resins made 3D printing increasingly feasible as well as popular in orthodontic clinics. These digital models, when printed using 3D printers, facilitate treatment planning as well as device fabrication, significantly boosting its utilization in dentistry, especially in the field of orthodontics [3-7].

Among the many available printers, the three famous desktop printing technologies are fused deposition modelling (FDM), SLA, and digital light processing (DLP), each implementing different as well as unique methods and materials for fabricating appliances [8].

Technologies and digital workflows in dentistry, which have been highlighted in recent literature, streamline procedures that offer superior appliance quality as well as faster treatment. Accurate dental models play a crucial role in diagnosis and are an integral part of all dental specialities. Thus, digital models produced using 3D printing present notable advantages over the traditional impression-taking and stone models [7].

However, along with accuracy, the mechanical properties of these printed models are of prime importance. Thus, evaluation of fundamental properties like flexure strength becomes crucial, as it ascertains the material's ability to withstand bending forces and having a high flexure strength means that the object can withstand stress-bearing procedures, like aligner making and appliance fabrication, which are used frequently in orthodontics [9]. Assessing this property of the 3D-printed models aims to elaborate their appropriateness as replacements to conventional plaster models, thus contributing to the ongoing transformation of conventional dentistry to digital dentistry [10].

Thus, this study aimed to assess the flexure strength of 3D-printed models using SLA, FDM, and DLP technology providing an insight into the sustainability and durability of 3D-printed dental models.

## Materials and methods

For this study, a total of 10 patient files were retrieved from the archives of a local orthodontic clinic, i.e., Bhatnagar Orthodontic and Dental Centre, Chandigarh, where the dental arches were scanned using Runyes® 3DS intraoral scanner (Zhejiang, China) and files were viewed in Nemotec software (Madrid, Spain) and evaluated.

Inclusion criteria included healthy periodontium, presence of maxillary first molar, and a minimum of 8 mm width of gingiva from the lowest part of marginal gingiva of the maxillary first molar.

Exclusion criteria included inadequately scanned posterior teeth, scans with overall quality that did not meet the expected standard, patients with compromised periodontal health, and patients with missing maxillary posterior teeth.

The three files were selected of the maxillary arches that met the inclusion criteria and were used for printing the models.

Dental models were fabricated using acrylonitrile butadiene styrene (ABS) polymer for the FDM printer and a polymethyl methacrylate (PMMA)-based liquid photopolymer resin material for both DLP and SLA apparatus printers. Table 1 gives the details of the printers and materials used.

**Table 1:** Detailed description of the materials and printers. Zortrex (Olsztyn, Poland); NextDent (Soesterberg, Netherlands); Formlabs (Somerville, MA); 3D Systems (Rock Hill, SC); Z Suite (Zortrex, Olsztyn, Poland); Sprint (3D Systems, Rock Hill, SC); PreForm (Formlabs, Somerville, MA). FDM: fused deposition modelling; DLP: digital light processing; SLA: stereolithography; ABS: acrylonitrile butadiene styrene; IPA: isopropyl alcohol; UV: ultraviolet.

	FDM	DLP	SLA
Manufacturer name of the printers	Zortrex	NextDent	Formlabs
Manufacturer name of the material used	Zortrex	3D Systems	Formlabs
Software used to view the STL files	3D Viewer	3D Viewer	3D Viewer
Software used to print the models	Z Suite	Sprint	PreForm
Material used for printing the casts	ABS	Photopolymer resin	Photopolymer resin
Orientation angle at which the casts were printed	Flat	45° angle	45° angle
Post-printing procedure (if followed)	None	IPA wash & UV cure	IPA wash & UV cure

Sample groups

Three distinct groups (n = 10) were formed. Group A included 10 models printed by a FDM printer. Group B included 10 models printed by a DLP printer. Group C included 10 models printed by an SLA printer.

Sample preparation for mechanical testing

**Figure 1:**
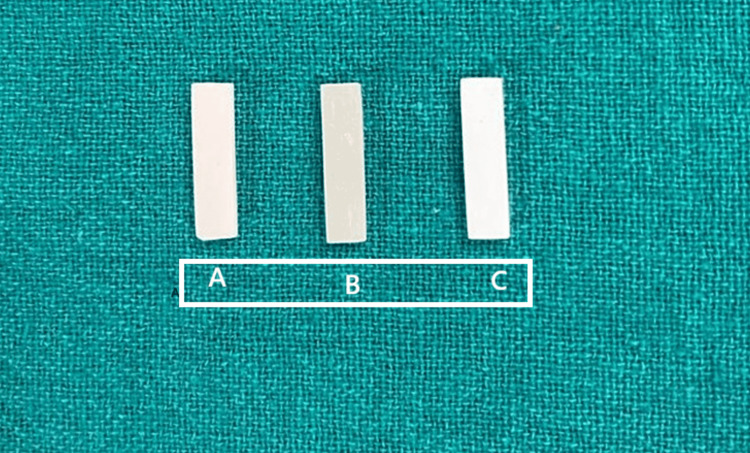
Prepared bars of 20 mm length, 5 mm width, and 2 mm height. A represents the bar cut out of models printed from an FDM printer, B represents a bar from a DLP printer, and C represents a bar cut out of a model printed from an SLA printer. Such bars of the same dimensions were cut out from all models printed from the different printers making 10 samples each from one printer. FDM: fused deposition modelling; DLP: digital light processing; SLA: stereolithography.

The printed models were marked 2 mm below the lowest point at the marginal gingiva in the maxillary first molar area. Bars measuring 20 x 5 x 2 mm were cut out from the printed models in the anteroposterior direction, creating 10 samples per printer group (Figure 1 shows the prepared blocks). These samples were then exposed to flexure strength testing using a three-point bending system.

**Figure 2:**
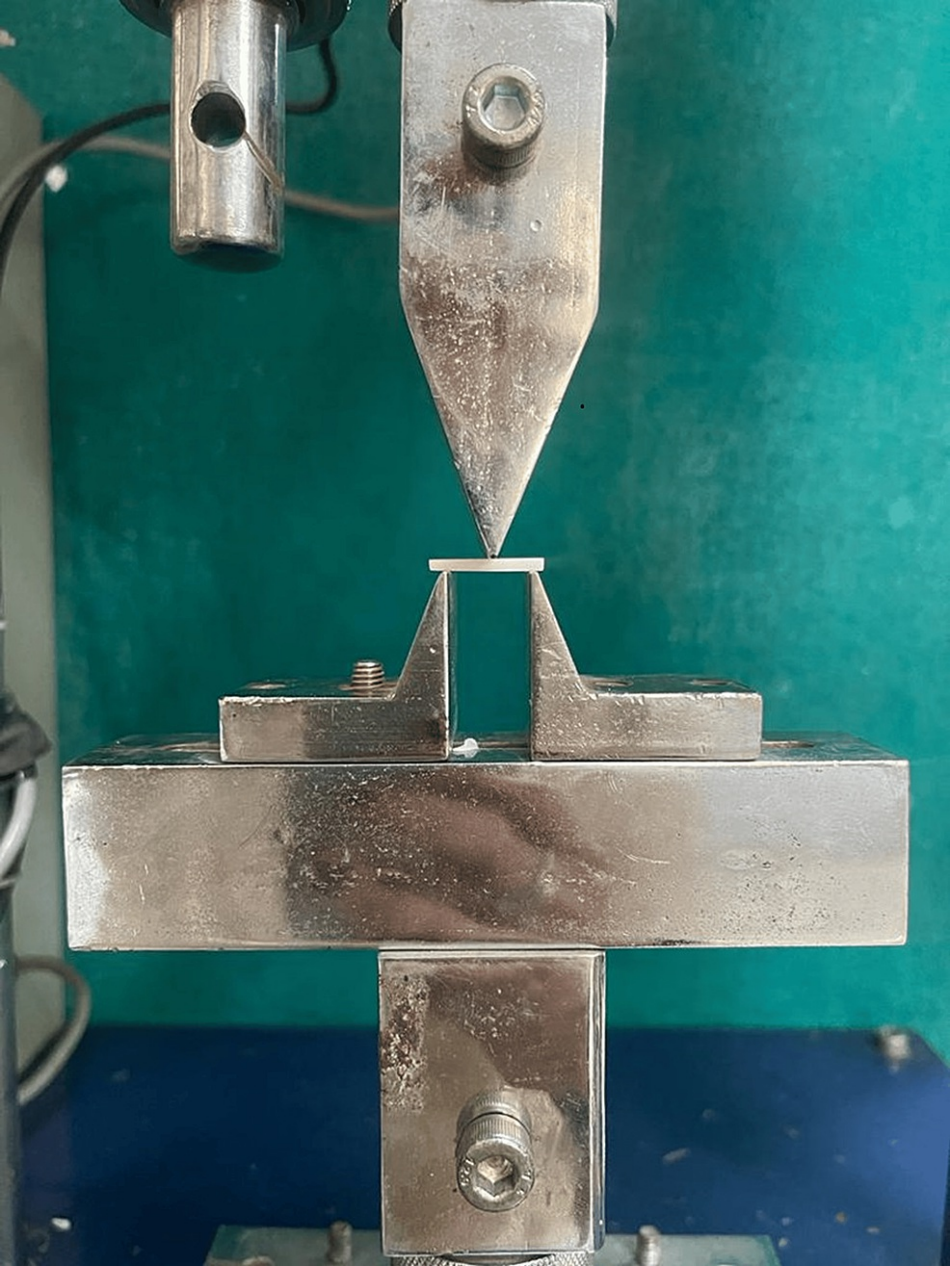
A schematic representation of the three-point bending test setup.

Flexure strength testing was carried out in accordance with the American Society for Testing and Materials (ASTM) D790 “Standard test methods for flexural properties of unreinforced and reinforced plastics and insulating materials” [10]. This method was chosen to attain some degree of a common denominator for the tests performed, in a reproducible and repeatable manner, on samples that were fabricated by different methods. A universal testing machine (Asian Test Equipments, Ravali, India; ISO 9001:2008) was utilized for the three-point loading system with two parallel supports employed to hold the bar and were subjected to an axial load at the centre with a cross head speed of 5 mm/min (Figure 2 shows the test set up). Flexural strength (σ- sigma) was calculated using the formula: $\sigma = \frac{3FL}{2bd^2}$. This formula represents the flexural strength (σ) in terms of the given variables: *F* for axial load, *L* for fracture point, *b* for the width of the sample, and *d* for the thickness of the tested specimen. The obtained flexure strength results were then subjected to statistical analysis.

## Results

In this study, the flexure strength of bars cut out from 3D-printed models obtained using FDM, DLP, and SLA apparatus printers was assessed. Statistical analysis, utilizing SPSS version 20.0 (IBM Corp., Armonk, NY), yielded mean values and standard deviations for force and flexure strength. A significance level of P < 0.001 was considered.

All samples tested had uniform length, width, and thickness and three groups (n = 10 each) represented FDM, DLP, and SLA printing technologies.

**Figure 3:**
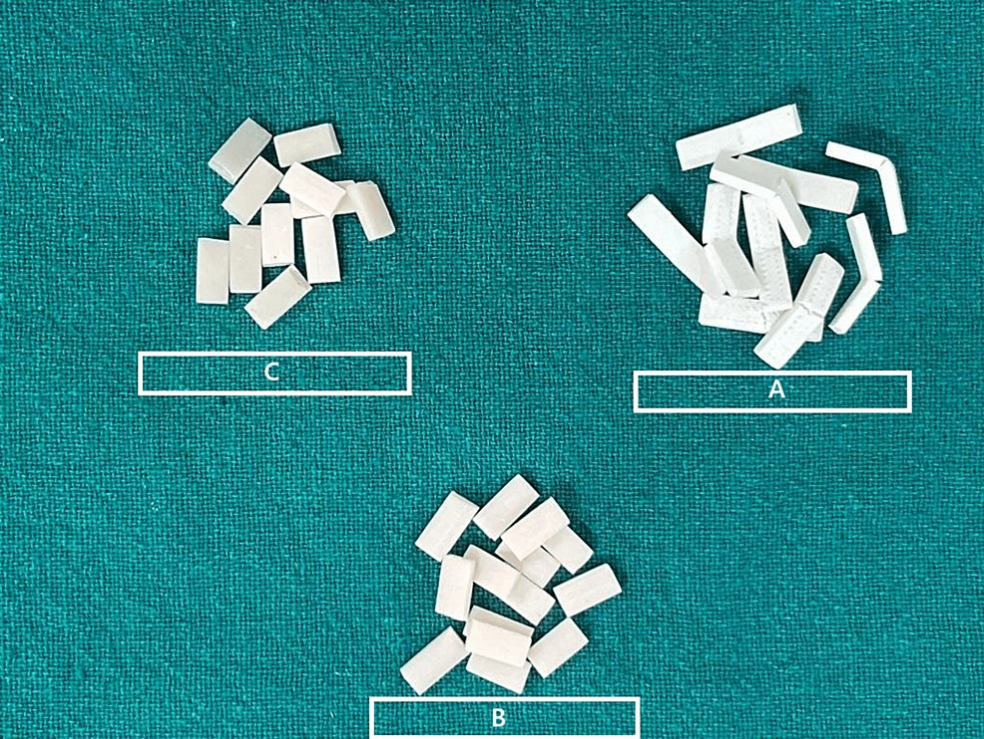
Fractured samples after being tested. Sample A represents the fractured samples from the FDM printer, B represents the fractured samples from the DLP printer, and C represents fractured samples from the SLA printer. All samples were subjected to an axial load of 5 mm/min at the centre until they fractured. FDM: fused deposition modelling; DLP: digital light processing; SLA: stereolithography.

All samples were subjected to an axial load at 5 mm/min at the centre until they fractured. Figure 3 shows the fractured samples.

Flexure strength and force value results are shown in Table 2 for the three groups where group A is the FDM group, group B represents the DLP group, and group C represents the SLA group.

**Table 2:** Representation of significant status force (N) and flexure strength (MPa). Group A is the FDM group, group B is the DLP group, and group C is the SLA group, with each group having 10 samples. The table represents maximum and minimum values of force and flexure strength amongst the three groups in Newton and megapascals, respectively. Intergroup comparison was performed using a one-way ANOVA test. It was revealed that results were found to be statistically significant for force and flexure strength for the three groups (P < 0.001). * Results are statistically significant, i.e., p < 0.001. FDM: fused deposition modelling; DLP: digital light processing; SLA: stereolithography.

Test variable	Group	Minimum	Maximum	Range	Mean ± SD	95% confidence interval for mean		P-value
						Lower bound	Upper bound	
Force	Group A	68.50 N	90.22 N	21.72	77.07 ± 6.7	72.273	81.864	<0.001*
	Group B	19.61 N	26.48 N	6.87	22.65 ± 2.38	20.953	24.355	
	Group C	41.19 N	106.89 N	65.70	67.47 ± 20.58	52.749	82.192	
Flexure strength	Group A	61.65 MPa	81.20 MPa	19.55	69.36 ± 6.03	65.046	73.677	<0.001*
	Group B	17.65 MPa	23.83 MPa	6.18	20.39 ± 2.14	18.855	21.920	
	Group C	37.07 MPa	96.20 MPa	59.13	60.72 ± 18.52	47.474	73.973	

The results of our study revealed that group A, i.e., the FDM group, exhibited significantly higher force and flexure strength than the other two groups. Group B, i.e., the DLP group, showed the lowest values, and their specimens fractured while the SLA group displayed higher flexibility when compared to the DLP group.

When the specimens manufactured using the same resin, which was in the case of the SLA and DLP groups, were compared, group C, i.e., the SLA group, demonstrated higher flexibility when compared to DLP, which could likely be linked to the difference in their printing methods. However, group A, i.e., the FDM group, showed the highest flexure strength, which could be because of a difference in the resin used.

## Discussion

This study judged the significant differences in force application and flexure strength among the three famously used 3D printers, i.e., FDM, DLP, and SLA.

Traditional orthodontics majorly relied on gypsum for dental models that presented challenges in storage as well as reproduction [7]. 3D printing addressed these drawbacks and offered many advantages like increased durability, lighter weight, and improved data-sharing capabilities [7].

In orthodontics, a significant reduction in the conventional alginate impressions and plaster models was seen with the introduction of 3D printing technology [1]. The accuracy and reproducibility of 3D-printed models have been highlighted in previous studies, suggesting their potential to help in accurate diagnosis, treatment planning, and appliance fabrication [1]. The present study tested the flexure strength, a mechanical property of these printed models, as it is a critical factor for applications like thermoforming, 3D metal printing, and surgical guide fabrication.

In a study conducted by Sang Mo Park et al., provisional restorations from different printers were tested and results showed that only the sample printed from FDM printers was dented and not fractured [3], showing that the FDM group has the highest flexure strength that aligns with the results of our study. Furthermore, the specimens printed using DLP mostly fractured into several pieces. The unique behaviour witnessed by the FDM group could be attributed to the ABS polymer resin, which has high elasticity [3]. In contrast, DLP and SLA groups, utilizing PMMA-based resins, demonstrated different behaviours, with DLP specimens exhibiting fractures at lower force values.

This distinction in results could be explained by the difference in the curing mechanisms of ultraviolet light in DLP and SLA technology, coupled with different surface morphologies [3].

Many studies have been conducted previously to check the dimensional accuracy. A study conducted by Patzelt et al. [11] on the dimensional accuracy of complete arch models containing 14 prepared teeth printed using SLA technology reported SLA to be better than the milled model, while in another study conducted by Kim et al. [12], a comparison of additively manufactured complete-arch orthodontic models was made, where the SLA group showed better trueness than DLP and FDM. Rebong et al. [13] in his study concluded that 3D-printed dental models made using SLA, PolyJet, and FDM statistically differ from the classic plaster models. Their results showed that PolyJet and SLA models showed a tendency towards expansion and shrinkage, while the models fabricated using FDM showed minor differences and could be a good replacement for plaster models. However, FDM printers using thermoplastics were not a requisite to laboratory processes like vacuum forming.

Though as per prior literature, the mechanical properties of acrylic used in 3D-printed models are considered to be exceptional, these models are not suitable for endless use. The acrylics used to print these models have the ability to absorb water, which could alter their dimensions [14]. Joda et al. in their study advised against the use of these models for fabricating definitive prosthetic reconstructions if the time period is longer than three to four weeks [14]. However, one must remember that the models can easily be reprinted from the available digital impressions, if the dimensions seem to be altered, irrespective of how much time has passed.

This study’s limitation lies in the fact that it tested only a subset of materials, whereas the extensive range available today offers the potential for a more comprehensive comparison of material strengths. Also, additional studies are required to check mechanical properties like fatigue strength, solubility, and permeability along with flexural strength. The physical properties of a resin are determined by many factors like flexural strength may change when the resin specimen is surrounded by a solvent [15]. The nozzle temperature of FDM can affect the bonding strength of the filaments. The printing speed and layer height also affect the tensile strength. These are to name only a few factors that affect the mechanical properties of samples [15].

The results of our study reveal that models printed using FDM showed great resistance against the bending force applied by the universal testing machine and showed an ample amount of fracture resistance. In clinical implications, it suggests that FDM models present a powerful alternative to traditional stone models in orthodontic practices. The ability to resist bending forces, coupled with the advantages of 3D printing technology, positions these models as valuable tools for record-keeping and patient care, which is in accordance with the study done by Marta Czajkowska et al. [7]. However, the study acknowledges the need for further research on additional mechanical properties, such as fatigue strength, solubility, and permeability, to comprehensively evaluate the performance and longevity of 3D-printed dental models.

## Conclusions

This study contributes valuable insights into the mechanical properties of 3D-printed dental models, emphasizing the impact of material and technology choices. It measured the flexure strength of models printed using FDM, DLP, and SLA technology. The ability of a material to resist bending forces is flexure strength and it plays an important role, as dental models whether printed or traditional should not only be accurate representations of the patient’s oral cavity but should also be fit enough to withstand forces that might be applied during different appliance fabrication.

The traditional stone models have proven to be accurate as well as mechanically fit for dental use; however, reproducing and storing these models has always been a tedious job. Thus, to join the technological wave as well as to overcome these drawbacks, 3D-printed models have been a good alternative to plaster dental models. Within the limitations of our study, the findings of our study support the suitability of FDM models for orthodontic applications, paving the way for enhanced efficiency and reliability in clinical practices.
